# Hierarchical DNA branch assembly-encoded fluorescent nanoladders for single-cell transcripts imaging

**DOI:** 10.1093/nar/gkac1138

**Published:** 2022-12-07

**Authors:** Xiaowen Cao, Feng Chen, Jing Xue, Yue Zhao, Min Bai, Yongxi Zhao

**Affiliations:** Institute of Analytical Chemistry and Instrument for Life Science, The Key Laboratory of Biomedical Information Engineering of Ministry of Education, School of Life Science and Technology, Xi’an Jiaotong University, Xianning West Road, Xi’an, Shaanxi 710049, P.R. China; Institute of Analytical Chemistry and Instrument for Life Science, The Key Laboratory of Biomedical Information Engineering of Ministry of Education, School of Life Science and Technology, Xi’an Jiaotong University, Xianning West Road, Xi’an, Shaanxi 710049, P.R. China; Institute of Analytical Chemistry and Instrument for Life Science, The Key Laboratory of Biomedical Information Engineering of Ministry of Education, School of Life Science and Technology, Xi’an Jiaotong University, Xianning West Road, Xi’an, Shaanxi 710049, P.R. China; Institute of Analytical Chemistry and Instrument for Life Science, The Key Laboratory of Biomedical Information Engineering of Ministry of Education, School of Life Science and Technology, Xi’an Jiaotong University, Xianning West Road, Xi’an, Shaanxi 710049, P.R. China; Institute of Analytical Chemistry and Instrument for Life Science, The Key Laboratory of Biomedical Information Engineering of Ministry of Education, School of Life Science and Technology, Xi’an Jiaotong University, Xianning West Road, Xi’an, Shaanxi 710049, P.R. China; Institute of Analytical Chemistry and Instrument for Life Science, The Key Laboratory of Biomedical Information Engineering of Ministry of Education, School of Life Science and Technology, Xi’an Jiaotong University, Xianning West Road, Xi’an, Shaanxi 710049, P.R. China

## Abstract

Spatial visualization of single-cell transcripts is limited by signal specificity and multiplexing. Here, we report hierarchical DNA branch assembly-encoded fluorescent nanoladders, which achieve denoised and highly multiplexed signal amplification for single-molecule transcript imaging. This method first offers independent RNA-primed rolling circle amplification without nonspecific amplification based on circular DNAzyme. It then executes programmable DNA branch assembly on these amplicons to encode virtual signals for visualizing numbers of targets by FISH. In theory, more virtual signals can be encoded via the increase of detection spectral channels and repeats of the same sequences on barcode. Our method almost eliminates nonspecific amplification in fixed cells (reducing nonspecific spots of single cells from 16 to nearly zero), and achieves simultaneous quantitation of nine transcripts by using only two detection spectral channels. We demonstrate accurate RNA profiling in different cancer cells, and reveal diverse localization patterns for spatial regulation of transcripts.

## INTRODUCTION

RNA in mammalian cells is generated by transcription of genomic DNA and matured by following processing events. Diverse RNA transcripts can be generated from the same DNA via sequence alternative splicing, nucleotide variation, epigenetic modification, etc (1–3). *In situ* RNA fluorescence imaging offers the opportunity to characterize their expression levels and subcellular localizations within cell samples or tissues. It is very important for studying gene regulation and cellular heterogeneity (4,5). Accurate representation of cell subtypes and states requires visualization of multiple transcript markers without nonspecific signals.

The targeted amplifying FISH was developed as a powerful tool to visualize individual transcripts in fixed samples. It can provide the copy numbers of both long and short transcripts with single-nucleotide resolution (6–10). Among them, rolling circle amplification (RCA)-assisted FISH is the most widely used method (9–12). It typically utilized one padlock probe or circular probe to recognize about 25-nt sequence of interested transcripts, and required another primer probe to initiate in situ RCA. Each long-strand amplicon can be visualized as one bright spot for digital quantitation. And the ideal RCA-assisted FISH should directly use target RNA as amplification primer without extra RNA treatment, and be scalable with multiplexing. In practice, existing methods do not meet these requirements. During past several years, we and others developed diverse DNAzyme-based signal amplification methods for in vitro biosensing and living-cell imaging (13–19). These DNAzymes are specialized DNA sequences that can catalyse RNA cleavage or other reactions (20–22). According to these achievements, we speculate that one circular probe integrated with DNAzyme sequence could initiate RCA without additional primers and RNA treatment. However, typical fluorophores suffer from broad spectrums and spectral overlap, resulting that only about four targets can be simultaneously detected by single round fluorescence imaging.

To promote the capacity of multiplexing, several emerging methods have been developed to achieve highly multiplexed RNA imaging by sequential rounds of DNA hybridization, imaging, and stripping (23,24). They can visualize hundreds of to thousands of transcripts in single cells depending on the round numbers. Nevertheless, the sequential imaging is very time-consuming (at least several days), quite complicated and highly error-prone. Besides spectrums, other optical information of fluorophores such as lifetime, anisotropy and intensity can be also utilized to encode different targets and increase multiplexing ability (25–28). In these fluorescent encoding, different DNA probes or assemblies are labelled with corresponding fluorophores to build fluorescent barcodes for programmable multiplexing. For example, SeqEA (23) achieved multiplexed tagging of RNAs by tuning DNA hybridization to change the fluorescence intensity of RCA amplicon. Therefore, encoding multiple signals in RNA-primed amplicons with different fluorescence characteristics could offer potential solution to design highly multiplexed and accurately amplified systems for transcript imaging. And the flexible and diverse design of DNA probes could offer space for different fluorescence characteristics.

Here, we report hierarchical DNA branch assembly-encoded fluorescent nanoladders, a denoised and highly multiplexed amplification method for single-molecule transcript imaging. This method mainly relies on three successive DNA assemblies. The first stage is RNA-primed RCA by DNAzyme-incorporated circular probes. This design eliminates nonspecific amplification noise resulted from additional primers, and can discriminate homologous sequences even with single-nucleotide variation. The second stage is programmable assembly of branched DNA barcodes on respective RNA-primed amplicons. These branched DNA barcodes mainly contain different sequences or different repetitive numbers of the same sequences for stable binding of corresponding fluorescent probes. They expand the design flexibility and diversity of DNA barcoding with different fluorescence characteristics. And the third stage is the assembly of fluorescent probes on branched DNA barcodes. In this way, we can use both fluorescence spectrum and fluorescence intensity for signal encoding. In principle, the multiplexing capacity can be scaled up by increasing the detection spectral channels and repeats of the same sequences. In this work, we take two detection spectral channels and three repeats as an instance, experimentally demonstrating nine multiplex imaging of transcripts. Finally, we validated our encoding strategy in fixed cells for simultaneously imaging of nine transcripts. And the nonspecific amplification noise in these samples was decreased from averaged single-cell 16 to nearly zero. The accurate quantification of multiple transcripts in single cells enables study of gene expression regulation on the spatial and functional organization of distinct cell types.

## MATERIALS AND METHODS

### Materials

All DNA oligonucleotides were purchased from Sangon Biological Co. Ltd. The sequences of these oligonucleotides are listed in supporting tables (Supplementary Tables S1–S4). Enzymes used in this work such as T4 DNA ligase, exonuclease I (*E. coli*), exonuclease III (*E. coli*) and Recombinant RNase Inhibitor (RRI) were purchased from Takara Biotechnology Co. Ltd. The phi29 DNA polymerase, T4 PNK, SplintR ligase and hot start Taq DNA polymerase were purchased from New England Biolabs Ltd. and DAPI was from Beyotime Biotechnology. All solutions were prepared using DEPC-treated water.

### Preparation of circular DNAzyme probes

The circular probes were prepared by a multi-step reaction including hybridization, ligation and digestion. The hybridization reaction was carried out in 20 μl 1 × T4 DNA ligase reaction buffer containing 12.5 μM padlock probes and 25 μM linker probes at 90°C for 10 min, followed by 70°C for 1 h, 55°C for 1 h and 37°C for 1 h. Then, 3 μl T4 DNA ligase was added and the ligation performed at 37°C for 2 h, followed by incubation at 65°C for 30 min to inactivate enzymes. Next, 5 μl Exonuclease I and 2 μl Exonuclease III were added to digest linear DNA in 50 ul reaction buffer at 37°C overnight. The enzymes inactivation performed by incubated the mixture at 80°C for 30 min. The resulting circularized probes were stored at –20°C.

### Real-time fluorescence and gel electrophoresis image of RNA-primed RCA

All *in vitro* RNA-primed RCA reactions were conducted at 37°C for 1 h in a volume of 10 ul containing 2 μl of 10 × phi29 DNA polymerase reaction buffer, 0.4 μl circularized probes, 2 μl primer (2 μM), 2 μl MgSO_4_ (100 mM), 3 μl dNTPs (2 mM), 2 μl 5 × sybre Green I, 0.25 μl T4 PNK and 0.25 μl phi29 DNA polymerase. The fluorescence intensity was recorded on LC 96 (Roche, Switzerland) in real time over a period of 30 min at 1 min intervals. Then the amplicons were monitored by 1% agarose gel electrophoresis, which was performed at 100 V for 40 min in 1 × TBE buffer.

### *In vitro* imaging of primed-RCA amplicons

Aminosilane-treated glass was prepared by incubating cleaned 22 × 60 mm glass coverslips for 1 min in 2% 3-aminopropyl triethoxysilane in acetone. A polydimethylsiloxane (PDMS) mask was applied to create a hybridization chamber (4 mm in diameter) on the slide. A blocking solution containing 10 ng/ml sonicated salmon sperm DNA, 2 × saline sodium citrate (SSC) buffer, and 0.05% Tween 20 was added, and the slide was incubated for 20 min at 37°C. Washing was performed once with 2 × SSC buffer. For hybridization of the amplicons, 3 mL of hybridization mixture containing 100 nM fluorophore-labeled detection probes, branched probes and primary probes in 2 × SSC buffer was added and incubated at 37°C for 30 min. After hybridization, the slides were ready for imaging.

### Hierarchical DNA branch assembly in single cells

Cell lines MCF-10A, MCF-7, MDA-MB231 and HeLa were cultured on a collagen-coated coverglass (22 × 60 mm) enclosed in a PDMS chamber (4 mm in diameter). The cells were fixed in 4% paraformaldehyde for 15 min at room temperature after they reached the desired confluency. Then the cells were permeabilized for 5 min with 0.5% v/v Triton X-100 at room temperature. The hybridization of the circular probe with the target sequences was carried out in a volume of 20 μl containing 0.4 μl circular probe, 1 μl DTT (100 mM), 0.5 μl tRNA (50 mg/ml), 0.5 μl RRI, 2 μl MgSO_4_ (100 mM), 2 μl 20 × SSC, 4 μl formamide and 9.6 μl DEPC-treated H_2_O overnight at 37°C. Wash samples three times with 2 × SSC for 3 min at room temperature.

The sample was then washed using 1 × phi29 DNA polymerase reaction buffer and RCA was performed for 2 h at 37°C with a 20 μl mixture containing 0.5 μl of phi29 DNA polymerase, 2 μl of 10 × phi29 DNA polymerase reaction buffer, 0.5 μl of T4 PNK, 5 μl of dNTP (10 mM), 0.5 μl of RRI and 11.5 μl of DEPC-treated H_2_O, followed by a wash using 2 × SSC. The branch probe hybridization reaction was conducted with 20 μl of reaction solution containing 2 μl of each FP probe (2 μM), 2 μl 20 × SSC, 1 μl DTT (100 mM), 1 μl sperm DNA (50 μg/ml), 4 μl formamide and DEPC-treated H2O for 45 min at 37 °C. Following a wash using 2 × SSC, the samples were mounted with 20 μl DAPI solution for 15 min at room temperature and then washed three times with 2 × SSC. The samples were ready for imaging.

### Hierarchical DNA branch assembly in tissue sections

Frozen mouse lung tissue sections were obtained from a commercial source PuHe (Wuxi, China). They were prepared by PuHe as following: the freshly dissected lungs were immersed into OCT and snapped frozen with liquid nitrogen. And the tissue block was sectioned into thickness of 10 μm and placed in the centre of a poly-l-lysine coated glass slide. Then the frozen slides were fixed in 4% paraformaldehyde for 20 min at room temperature. Afterward, hierarchical DNA branch assembly was performed as described in fixed cells with little modification. Briefly, the total volume rises from 20 to 50 μl.

### Image acquisition and analysis

Fluorescence imaging was performed using a Leica TCS SP8 inverted confocal microscope (Leica, Germany). The images of RCA amplicons were acquired using a 63 × water-immersion objective. The DAPI dye was excited with a 405 nm laser line and detected with a 410–480 nm band-pass filter. The Alex 488 was excited with a 490 nm laser line and detected with a 495–545 nm. The Alex 555 was excited with a 552 nm laser line and detected with a 545–590 nm band-pass filter. The Cy5 was excited with a 649 nm laser line and detected with a 655–700 nm band-pass filter. Images were collected as z-stacks to ensure that all RCA amplicons were imaged. Stacks of images were taken with 500 nm between the z-slices, and combined to a single image by maximum intensity projection (MIP) using LAS AF Version. The images were processed by ImageJ version 1.46r software.

### Real-time quantitative PCR (RT-qPCR) analysis of mRNA inside cells

Total RNA of HeLa cells was extracted using TriZol (Thermo Scientific) following the manufacturer's instructions. The cDNA samples were prepared using RevertAid First Strand cDNA Synthesis Kit (Thermo Scientific, K16225) as the manufacturer's instructions. Then cDNA samples were stored at -80°C for future use. RT-qPCR analysis of mRNA was performed with a total volume of 50 μl reaction solution contained 2 μl of cDNA sample, 1 μl hot start Taq DNA Polymerase (1 U/μl), 5 μl 10 × reaction buffer, 5 μl forward primer (2 μM), 5 μl reverse primer (2 μM), 5 μl dNTP (2 mM) and 29 μl DEPC-treated water. The qPCR conditions were as follows: annealing at 94°C for 3 min, then followed by 50 cycles of 94°C for 30 s, 58°C for 30 s and 72°C for 45 s. Ct values were converted into absolute GAPDH copy numbers using a standard curve from a control RNA (human GAPDH mRNA in RevertAid First Strand cDNA Synthesis Kit). Every experiment was repeated three times. The copy number of other target mRNAs was evaluated by referring to the expression of GAPDH mRNA using the 2^–ΔΔCt^ method.

## RESULTS

### Overview of hierarchical DNA branch assembly-encoded fluorescent nanoladders

**Figure 1.**
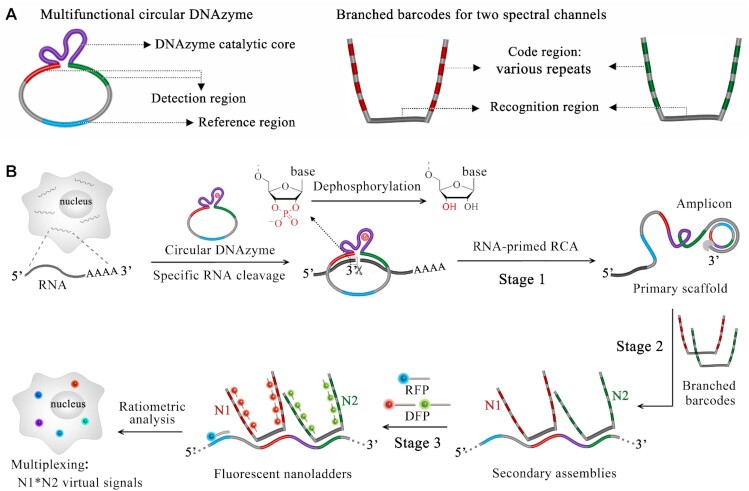
(**A**) Design of circular DNAzyme and branched barcodes. (**B**) Overview of hierarchical DNA branch assembly-encoded fluorescent nanoladders for single-cell transcripts imaging. *N*: the numbers of repetitive sequence. RFP/DFP: reference/detection fluorescent probe.

The hierarchical DNA branch assembly-encoded fluorescent nanoladders are constructed via three sequential processes: the RNA-primed RCA using a circular DNAzyme to produce the long single-stranded amplicon, the programmable assembly of branched barcodes on this RCA amplicon and the final assembly of fluorescent probes on branched barcodes (Figure 1). The circular DNAzyme is designed to contain a DNAzyme catalytic core (purple part), two recognition arms (red and green part, 20 nt each) and a reference region (blue part, 20 nt). The reference region is designed to normalize the different signal intensities due to the various sequence lengths of RCA amplicons. The two recognition arms enable the specific hybridization to the interested RNA. In this circular DNAzyme/RNA assembly, an RNA dinucleotide kink is formed at the junction. In the presence of divalent cations such as Mg^2+^, the DNAzyme is activated and then cleaves the RNA substrate at this dinucleotide site. The upstream 5’-cleaved product contains a 2’, 3’-cyclic phosphate at its 3’-end. The following dephosphorylation treatment generates a hydroxyl group at this 3’-end. Thus,this RNA cleaved product can work as the primer to initiate *in situ* RCA namely RNA-primed RCA. In this way, the additional primer as well as its nonspecific amplification will be avoided.

For subsequent two-step programmable assembly on RNA-primed amplicons to form the fluorescent nanoladders, series of branched barcodes and fluorescent probes (detection or reference) are designed. Each amplicon is repetitively hybridized with one or more branched DNA barcodes and one reference fluorescent probe (RFP). The branched DNA barcodes have the code region for stable hybridization with detection fluorescent probes (DFP, length = 20 nt). The code regions are designed as various repeats of same sequence. The fluorescent nanoladders can be visualized as bright spots with different fluorescence intensities and thus be identified as corresponding RNA transcripts. More virtual signal channels can be encoded by increasing the detection spectral channels and repeats. It is well known that the RCA efficiency is not uniform especially in molecularly crowded intracellular environments or at solid–liquid interfaces. The RFP was employed to normalize the signals. Thus, final virtual signals are obtained by ratiometric analysis (detection channel/reference channel).

### Validation of circular DNAzyme-enabled RNA-primed RCA

**Figure 2.**
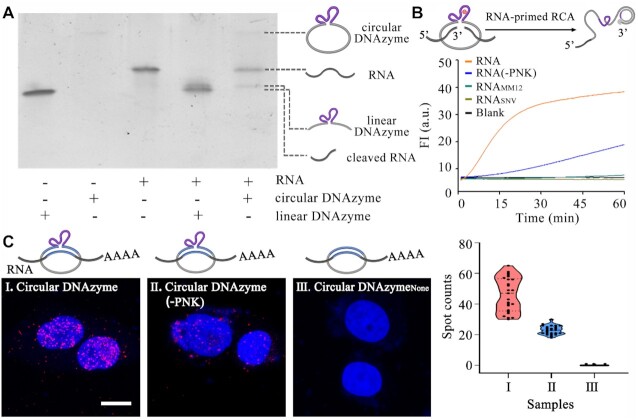
Validation of circular DNAzyme-enabled RNA-primed RCA. (**A**) Electrophoretic analysis of cleavage products by linear and circular DNAzyme. Substrates and products were resolved by 15% denaturing PAGE. (**B**) Real-time fluorescence of RCA with circular DNAzyme in different cases. (**C**) Left: representative images of target RNA in single cells using the circular DNAzyme-enabled amplification (red, Cy5 for GAPDH; blue, DAPI; MDA-MB-231 cells were used here). The scale bar is 10 μm. Right: statistical analysis of amplicon spot counts for each sample in single cells (*N* = 20). *N* represents the number of single cells for each sample.

Firstly, the preparation of circular DNAzymes and their RCA feasibility were verified by gel electrophoresis. In brief, the circular DNAzymes are formed by the ligation of linear DNAzymes on single-stranded probes. After ligation, DNA Exonuclease I and Exonuclease III are added to digest residual linear probes. As shown in Supplementary Figure S1, there is no obvious by-products except the circular DNAzymes in the relevant gel lanes. And they can act as templates to start RCA with a primer. Then, the RNA cleavage capacity of the circular DNAzymes was investigated by using a model RNA substrate. As shown in Figure 2A, the circular DNAzyme cleaves RNA with the efficiency only slightly lower than that of a canonical linear DNAzyme. It is well known that the RNA 5’-cleaved product by DNAzyme contains one base overhang with 2’,3’-cyclic phosphate at its 3’-end. And the phi29 DNA polymerase has an inherent 3’ to 5’ exonuclease activity. Therefore, three primers with different 3’-ends were designed to assess the RCA efficiency. As showed in Supplementary Figure S2, the RCA products initiated by Pr3 (with both one base overhang and 3’-phosphate) is lower than those by Pr1 (without any 3’ modifications) and Pr2 (with one base overhang). Compared to phi29 DNA polymerase, other DNA polymerases without 3’ to 5’ exonuclease activity result in much lower RCA signals when using this Pr2 primer (Supplementary Figure S3).These results indicate that the base overhang with hydroxyl group can be well removed by phi29 DNA polymerase, and the phosphate group at 3’-end of primer hinders the RCA reaction. To remove the phosphate group at 3’-terminal of cleaved RNA, we employed T4 PNK in our strategy. The real-time fluorescence analysis was used to investigate the RCA efficiencies with PNK or without PNK. As shown in Figure 2B, the fluorescence signal increased fast and reached higher value with PNK in RCA system. Meanwhile, we assessed the RNA cleavage specificity by the circular DNAzymes. The target RNA is designed with two regions (20nt each) complementary to the circular DNAzyme. Two variants of RNA substrates are designed as negative controls, including an RNA variant with 12-nucleotide mismatch (RNA_MM12_) and a single-nucleotide variant (SNV) with the mismatched nucleotide in the middle (RNA_SNV_). These mismatched RNA substrates are disabled to initiate the cleavage reaction and the following RCA (Figure 2B). Similar results were obtained by the linear DNAzyme (Supplementary Figure S4). These results confirmed the excellent performance of our RNA-primed RCA *in vitro*.

**Figure 3.**
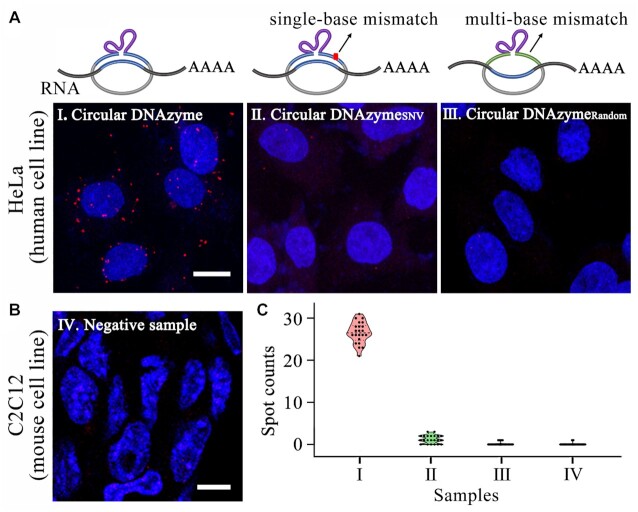
Demonstration the specificity of our method *in situ*. (**A**) Representative cell images of THBS1 mRNA in human cell HeLa with circular DNAzyme, circular DNAzyme_SNV_ and circular DNAzyme_Random_. (**B**) Representative cell images of mouse cell C2C12 with circular DNAzyme probe designed for human THBS1 mRNA. (**C**) Statistical analysis of amplicon spot counts for each sample in single cells (*N* = 20). *N* represents the number of single cells for each sample. Red, Alexa 555 for THBS1; blue, DAPI. The scale bars are 10 μm.

Next, the imaging experiments in fixed cells were performed by our method. The human GAPDH mRNA is chose as the target transcript here. As a negative control, the defective circular probe without DNAzyme core sequence (circular DNAzyme_None_) is designed. As shown in Figure 2C, the circular DNAzyme_None_ indued nearly no fluorescent signals in the cells. In contrast, bright fluorescent spots were observed in the cells treated with the circular DNAzyme for GAPDH mRNA. Especially, the addition of PNK treatment offered more spots in these cells. These *in situ* imaging results demonstrated the requirement of DNAzyme-catalyzed RNA cleavage and 3’ dephosphorylation for our method, which is consistent with those of the *in vitro* analysis mentioned above. Moreover, we applied our method to imaging human THBS1 mRNA for the investigation of the detection specificity in fixed cells. Besides the circular DNAzyme matched to THBS1 mRNA, two defective circular DNAzyme probes are used. One contains single-base mismatch to the target sequence of THBS1 mRNA (termed circular DNAzyme_SNV_), and the other one has random sequence that is not complementary to THBS1 mRNA (termed circular DNAzyme_Random_). As shown in Figure 3, the number of spots in single cells by these two mismatched probes ranged from zero to three. These values were far much lower than those by the matched circular DNAzyme. Furthermore, we used this matched probe for human THBS1 mRNA to test the mouse C2C12 cell line as a negative sample. The sequence of THBS1 mRNA in the mouse C2C12 cells is quite different to that in human cells. We observed nearly no spots in the C2C12 cells (Figure 3). All these results demonstrated that our method is highly specific to interested RNA in fixed cells without remarkable nonspecific amplification.

Notably, the rare nonspecific spots by our method may suffer from off-target detection of abundant endogenous RNA molecules or unpurified pre-prepared circular DNAzyme. An additional *in situ* circularization of linear DNAzyme by enzymatic ligation may possibly eliminate these rare occasional false signals, which were well confirmed in previous works (29,30). For a further study, we used *in situ* circularization of linear DNAzyme instead of the pre-prepared circular DNAzyme in our method as a control experiment. As shown in Supplementary Figure S5, the RNA detection efficiency decreased by the additional *in situ* circularization. As we know, the diffusion and reaction efficiency of enzyme molecules and DNA probes may be limited in the crowded intracellular environment (31,32). More *in situ* enzymatic reactions could offer lower detection efficiency. The optimization of ligation reaction conditions can further improve the detection efficiency. In our work, the design of circular DNAzyme provides high sequence specificity with SNV discrimination and very low nonspecific amplification. Thus, we do not require the *in situ* circularization of linear DNAzyme probes in our method.

### Comparation of RNA-primed RCA with additional primer-assisted RCA

**Figure 4.**
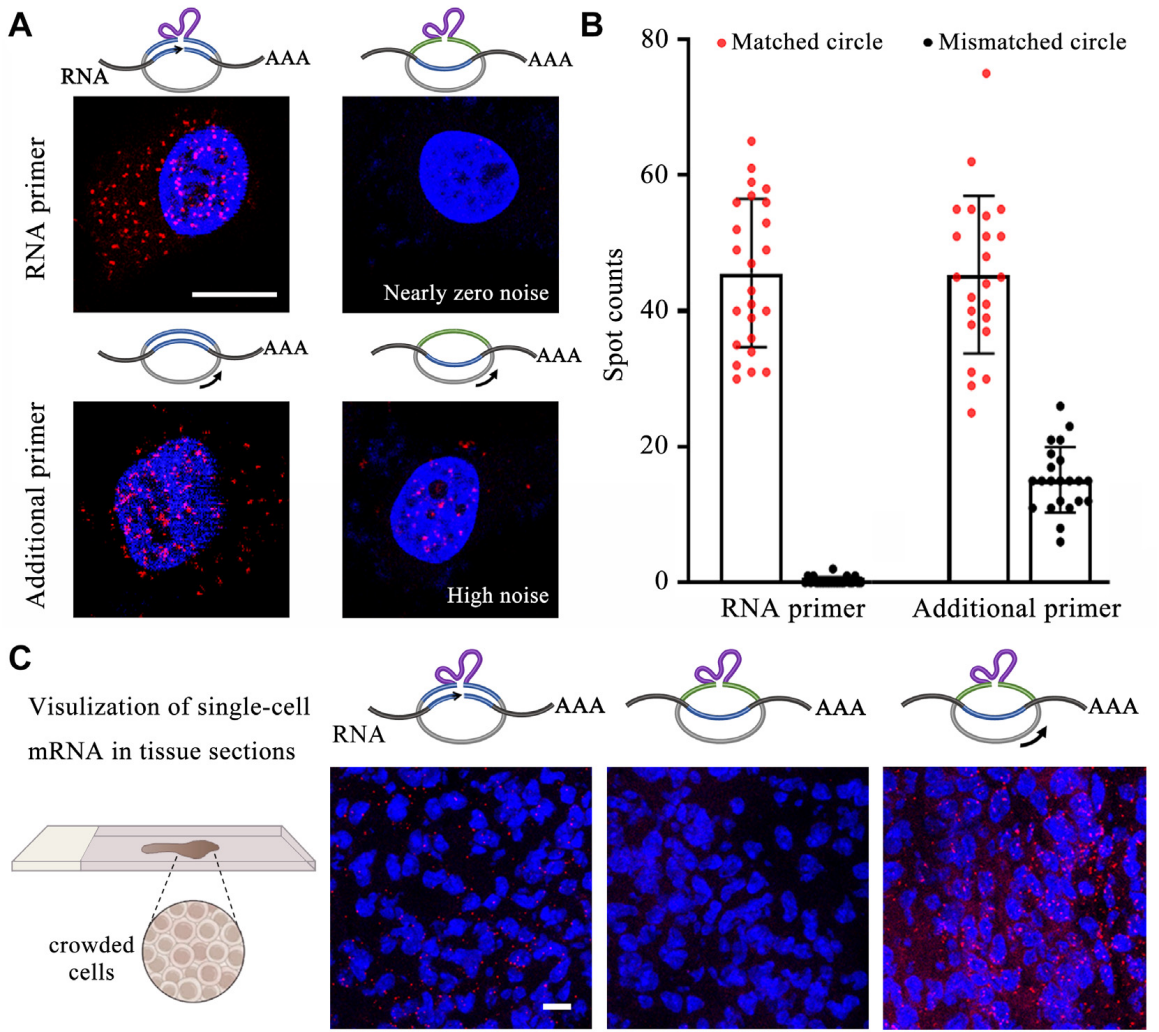
Comparation of RNA-primed RCA with additional primer-assisted RCA. (**A**) Representative cell images of human cell MDA-MB-231 by both methods using matched and mismatched circle probes (red, Cy5 for GAPDH; blue, DAPI). (**B**) Statistical analysis of amplicon spot counts for each sample in single cells (*N* = 25). *N* represents the number of single cells for each sample. (**C**) Representative images of mouse lung tissue sections using RNA-primed RCA with different probes (red, Alexa 555 for THBS1; blue, DAPI). The scale bars are 10 μm.

In traditional *in situ* RCA for amplifying RNA FISH, both a circular template and an additional primer are used together. The off-target hybridization or the nonspecific adsorption of these DNA probes may cause serious false positive signals. In contrast, our proposed RNA-primed RCA achieved nearly no nonspecific amplification as mentioned above. For a comparative analysis, we detected the fixed samples (cell lines and tissue sections) by these two types of *in situ* RCA method, respectively. Firstly, we detected the GAPDH mRNA in MDA-MB-231 cells using defective circular probes as negative controls. They contain several-base mismatch to the target sequence of GAPDH mRNA. As shown in Figure 4A, the spot count of single cells was used for the quantitative analysis. A range of 6–26 spots was observed in the cells by the additional primer-assisted RCA with the mismatched circular probe, indicating the remarkable nonspecific amplification. Oppositely, nearly zero nonspecific spots were caused by our RNA-primed RCA. On the other hand, these two methods presented a comparable detection efficiency. Subsequently, the tissue sections were detected by these two methods. It is well known that the tissue samples present higher nonspecific adsorption than cell samples. The THBS1 mRNA in mouse lung tissue sections was analyzed here. It can be seen in Figure 4B that the additional primer-assisted RCA suffered from very severe false positive fluorescence signals. Our method still offered practicable detection efficiency and high specificity. The excellent performance will enable its application in the multiplexed imaging.

### Demonstration of the multiplexing capacity of fluorescent nanoladders with one detection spectral channel

**Figure 5.**
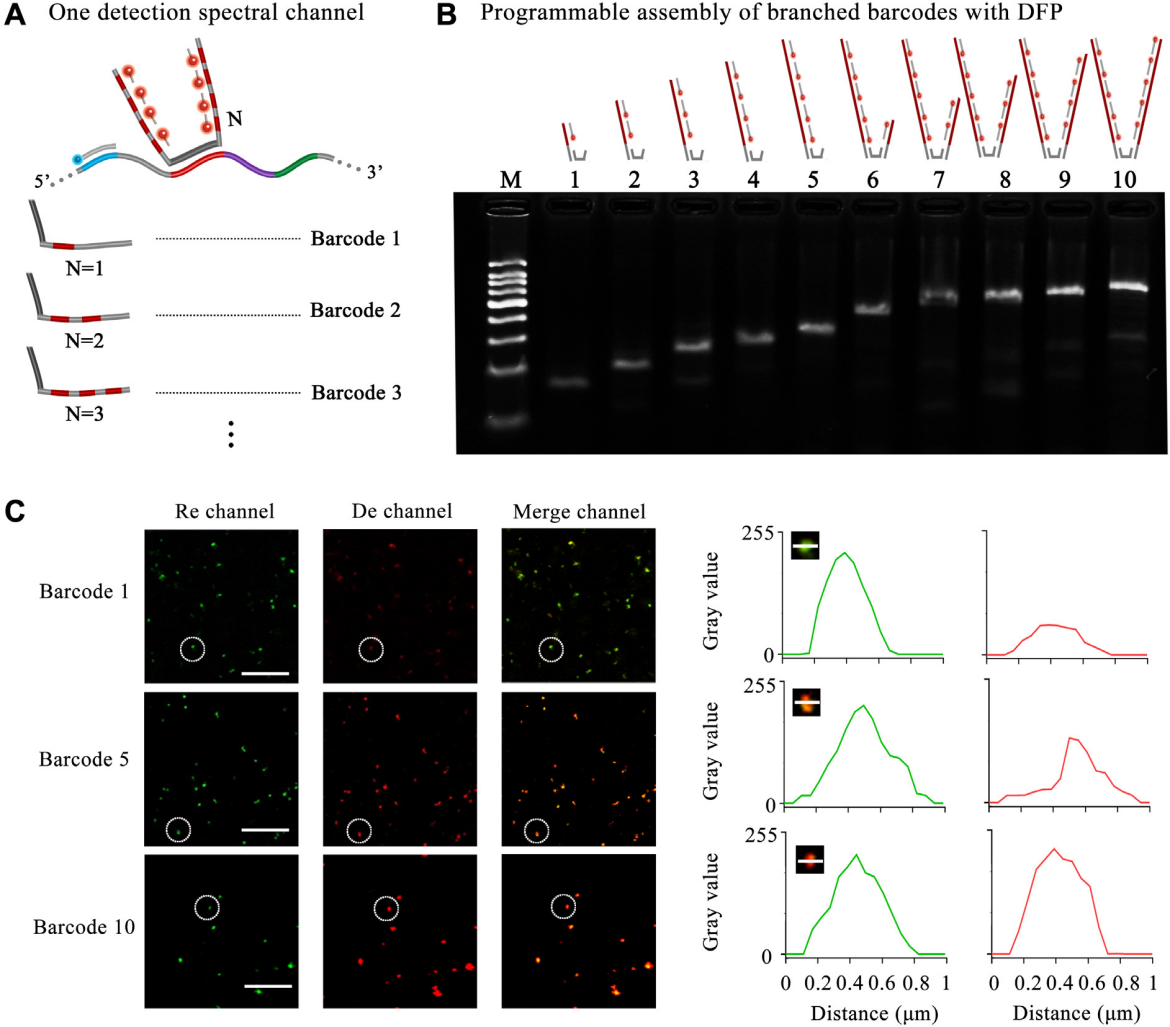
The multiplexing capacity of hierarchical DNA branch assembly-encoded fluorescent nanoladders with one detection spectral channel. (**A**) Schematic representation of different fluorescent nanoladders. (**B**) Analysis of programmable assembly of DNA barcode duplexed with different number of DFPs (labelled with Cy5). The assemblies were resolved by 3.5% agarose gel. The M means 50 bp DNA ladder. (**C**) Left: the fluorescence images of amplicons; scale bars: 10 μm. Right: the image of a single amplicon spot (top left) and the line scans of gray value in the Re and De channels corresponding to the white line through the single amplicon spot. The x axis represents the relative position of the pixels in the white line, and the y axis is the gray value of the pixels.

To increase the multiplexing capacity, the RCA amplicons were encoded by DNA branch assembly in our method. And we chose one detection channel as instance. As shown in Figure 5A, the number of DFP hybridized to branched barcodes on amplicons can be determined by the repeats of the same sequence. To simplify the design, we first prepared the DNA barcode duplexes by the hybridization of the branched barcodes to the two terminal overhangs of the bridged DNA probes (Figure 5B). Ten branched barcodes were used here, offering the number of DFP from 1 to 10. Gel electrophoresis was used to analyze the yield of different assembly barcodes with DFPs. All ten different architectures were successfully assembled with high yield (Figure 5B, Supplementary Figure S6). It is demonstrated that the branched DNA design can increase the number of barcodes to tag the target sequences. These assemblies of branched barcodes with DFPs can be programmble for multiplexed fluoresecence imaging.

In the multiplexed design, different pre-hybridized DNA barcode duplexes are simultaneously added for the branched assembly. They should not disturb each other via the competitive hybridization at the terminal overhangs of the bridged DNA probes. To evaluate the unwanted reaction, we chose barcode 2 and barcode 5 as an example. We found that excessbarcode 5 did not disturb the pre-hybridized DNA barcode 2 duplex (Supplementary Figure S7). This enabled the accurate assembly of multiplex branched barcodes in our method. Beside the detection spectral channel, a reference spectral channel is employed to normalize the changes of sequence length of amplicons. It is well known that the RCA efficiency is not uniform especially in the molecularly crowded intracellular environment. To demonstrate the multiplexing capacity of our method, we encoded three virtual signal channels by the assembly of different numbers of DFP and the same RFP to respective RCA amplicons. In the *in vitro* experiment, the RCA amplicons were first captured on the coverglass, followed by those assemblies. After that, the fluorescence imaging analysis was performed (Figure 5C). The assembled amplicons were visualized as bright spots in both spectral channels. It can be seen in Supplementary Figure S8 that there was no fluorescence resonance energy transfer effect between two fluorophores assembled on the RCA amplicons. The fluorescence intensities of spots in De channel increased from barcode 1 to barcode 10, depending on the numbers of DFP molecules. Especially, different spots of the same virtual signal channel presented relatively wide distribution of fluorescence intensities (Supplementary Figure S9). This result mainly caused by the nonuniform RCA efficiencies and amplicon lengths. The normalization of the fluorescence intensities in the De channel to those in the Re channel help to reduce the deviation. Notably, the theoretical fluorescence intensity ratio of barcode 1 was expected as 0.1 in this design of virtual signals. However, the really detected ratios were distributed from 0 to 0.2 (Supplementary Figure S9). This deviation between detected fluorescence signals and the theoretical values mainly suffer from the heterogeneous assembly of both branched barcodes and fluorescent probes with amplicons at solid-liquid interfaces. Pre-assembled branched barcodes and DFP as fluorescent barcodes with purification could help to reduce the deviation. Similar results have been *in vitro* demonstrated in previous works (33). Yet, the reaction efficiency in crowded intracellular environments may by still restricted. Nevertheless, the branch design expands the flexibility and diversity of DNA barcoding with different fluorescence characteristics. In theory, the addition of other fluorescence characteristics such as anisotropy would further increase the multiplexing capacity of our method. Besides *in vitro* analysis of the multiplexing barcoding, a transcript in fixed cells was detected by these three virtual signal channels (Supplementary Figure S10). Similar results about signal intensity were obtained.

### Multiplexed RNA imaging in single cells by hierarchical DNA branch assembly-encoded fluorescent nanoladders

**Figure 6.**
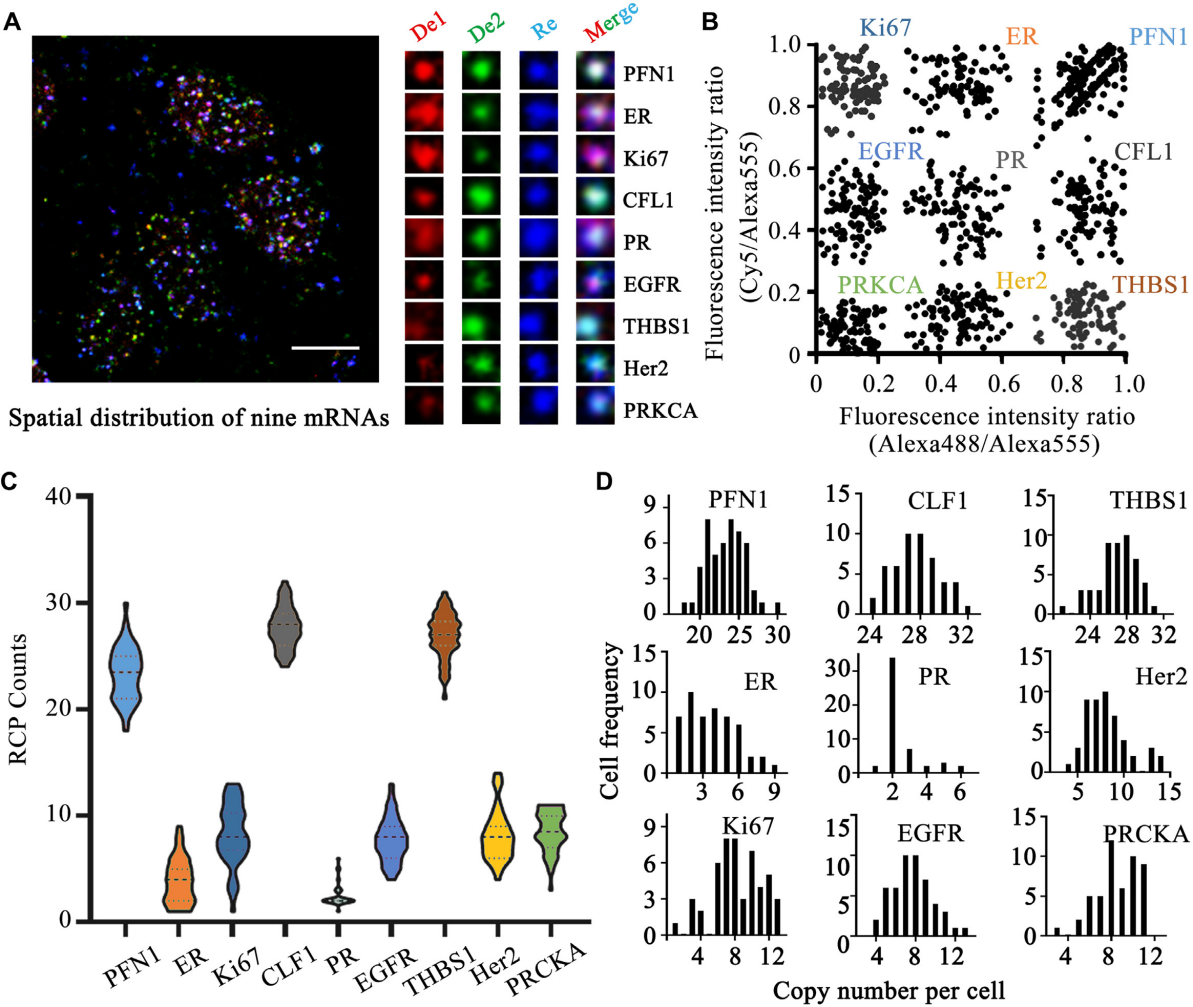
Multiplexed RNA imaging in single cells by hierarchical DNA branch assembly-encoded fluorescent nanoladders with two detection spectral channel. (**A**) Representative cell image for the spatial distribution of the nine transcripts in HeLa cells. (**B**) Histogram plots for the fluorescence signals of the nine encoded amplicons. (**C**) The statistical analysis of nine target transcripts in single cells. (**D**) Histograms of the copy number of nine target transcripts.

Based on above results, we employed two detection spectral channels to encode more virtual signals in fixed cells (Supplementary Figure S11). And it shown that the spots in all three channels (detection and reference channels) were colocalization, indicating that the feasibility of two detection channels encoding. We then explored the method for multiplexed imaging of transcripts in HeLa cells. For a proof of principle experiment, we designed different circular DNAzymes and branched probes for the detection of nine transcripts in single cells. The sequences of these probes are described in Supplementary Table S3. As mentioned above, *in situ* RNA-primed RCA enabled by circular DNAzymes was performed in the fixed cells, followed by the branched assemblies. It can be seen in Figure 6A that the amplicons of different target transcripts were marked with different pseudo colors. Plotting the virtual ratiometric channels distribution of the amplicons (channel De 1/Re and De 2/Re) demonstrated that the nine kinds of amplicons targeting different transcripts were separated (Figure 6B), indicating that the multiple target mRNAs could be identified and encoded by our method. We further investigeted the variability in the expression level of each measured gene in single HeLa cells (Figure 6C and D). We revealed sinificanct cell-to-cell variation in expression for these transcripts. In brief, the detected copy numbers of PFN1 in single cells varied from 18 to 30. And the ER presented a low expression level with 1–9 copies in single cells. More systematic analysis of these single-cell RNA expression levels may allow the accurate representation of cell subtypes and states. On the other hand, we studied the detection efficiency of our method by a comparison with RT-qPCR data. We detected THBS1, Her2 and PR in HeLa cells as examples. The detection efficiencies for these three transcripts were 28.6%, 30.1% and 29.3%, respectively (Supplementary Table S5). These detection values are consistent with those of previously reported methods such as ISS (34) and comparably higher than those *in situ* reverse transcription-based RCA methods (10).

**Figure 7.**
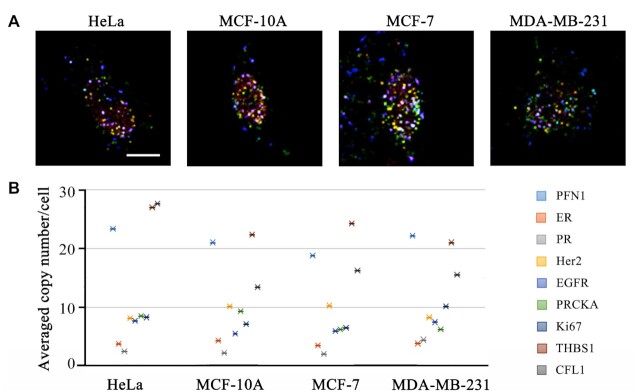
Multiplexed RNA imaging in different cell lines. (**A**) Representative cell image for the spatial distribution of the nine transcripts in different cell lines. (**B**) The cell discrimination model established using nine transcripts data.

Moreover, we used our multiplexed imaging method to detect these nine transcripts in different cancer cell lines. As shown in Figure 7, nine transcripts in these cell lines showed diverse expression levels and subcellular distribution. In brief, the averaged copy number of PR, EGFR or Ki67 in MDA-MB-231 is higher than that in MCF-7 or MCF-10A, possibly implying that these gene might be related to the breast cancer metastasis. On the other hand, the averaged copy number of PRKCA in MCF-10A is higher than that in the other three cell lines, indicating the potential negative effect of PRKCA in cancer cells. We observed no remarkable changes in the copy number of ER in these four cell lines.

To further increase the multiplexing capacity of our method, one simple strategy is to increase the numbers of repetitive sequences on the branched DNA barcodes. However, the commercially available DNA probes are limited to a length of up to about 150–200 mer. More seriously, the wide overlaps of fluorescence intensities have been observed in different virtual signals. These overlaps restricted the numbers of practically available signal channels as mentioned above. Another strategy is the sequential rounds of DNA hybridization, imaging, and stripping, which has been well demonstrated in highly multiplexed FISH methods. In this work, we also performed a proof-of-concept experiment by using our method with two rounds of these imaging processes. As shown in Supplementary Figure S12, the fluorescence signals for different transcripts in the first-round imaging were well removed, and those in the second-round imaging were bright. These results hold the potential for the imaging of 18 (9 + 9) and even more transcripts in single cells. Despite the proof of concept data, more systematic assessments are required for the highly multiplexed version of our method.

## DISCUSSION

In summary, we demonstrated a DNA-encoded amplifying imaging method, hierarchical DNA branch assembly-encoded fluorescent nanoladders, that enables maximally denoised and highly multiplexed visualization of single-cells transcripts. Our method mainly relies on *in situ* RNA-primed RCA and subsequent branch assembly-encoded fluorescence imaging. It nearly eliminates nonspecific amplification, and expands DNA barcoding space on amplicons. As a proof-of-concept demonstration, we designed programmable branch assemblies of different numbers of the same fluorescent probes on respective amplicons to create virtual signal channels. The amplicons visualized with different fluorescence intensities are identified as different transcripts. In theory, more virtual signal channels can be encoded by increasing the detection spectral channels and the repeats of same fluorescent probes. We also use a reference spectral channel to normalize the sequence length difference in the same amplicons. In this work, we experimentally demonstrating 9 multiplex imaging of RNA. Finally, this method was applied to fixed cells. The nonspecific noise in these samples were decreased from averaged 16 to nearly zero. The copy numbers and distribution of nine transcripts in single cells were simultaneously detected via one round fluorescence measurement. The accurate quantification data of multiple transcripts enabled study of regulation of gene expression on the spatial and functional organization of distinct types of cells.

Besides, the intracellular environments would limit rapid diffusion of large-size DNA probes. Thus, the hybridization efficiencies between amplicons and branched barcodes or RFP may be varied depending on their lengths and structures. The difference in hybridization efficiencies may cause deviation between detected fluorescence signals and the theoretical values. Pre-assembling branched barcodes and DFP as fluorescent barcodes with purification could help to reduce the deviation (33). Yet the reaction efficiency in crowded environments is still restricted. It is an open problem in cellular imaging. Nevertheless, the branch design expands the flexibility and diversity of DNA barcoding with different fluorescence characteristics. In theory, the addition of other fluorescence characteristics such as anisotropy would further increase the multiplexing capacity of our method.

Our hierarchical DNA branch assembly-encoded fluorescent nanoladders can present better improvement for tissue samples that cause worse nonspecific adsorption and off-target amplification of DNA probes. We further envision that the addition of other fluorescence characteristics such as anisotropy would further increase the multiplexing capacity of our method. Despite the achievements, several open challenges still exist. Firstly, the RCA efficiency is not uniform in molecularly crowded intracellular environments. The different sequence lengths of the same amplicons may confuse the fluorescence intensities, though one RFP is used to realize ratiometric analysis. The intracellular environments also limit rapid diffusion of large-size DNA probes. Thus, the hybridization efficiencies of amplicons to branched barcodes or RFP may be varied depending on their lengths and structures. The difference in hybridization efficiencies may cause deviation between detected fluorescence signals and the theoretical values. We can pre-assemble branched barcodes with DFP and RFP to form ratiometric fluorescent barcodes with purification. These purified ratiometric fluorescent barcodes could help to reduce the deviation from different hybridization reactions. Yet, the reaction efficiency in crowded environments may still be a problem. In addition, the spatial overlap of multiple amplicons or optical crowding often exist in intracellular environments, especially for highly multiplexed detection or imaging high-abundance transcripts (35). The development of super resolution microscopy (36) and expansion microscopy (37) may overcome the optical crowding, enabling super-resolved profiling of transcriptome.

## DATA AVAILABILITY

The data underlying this article are available in the article and in its online supplementary material.

## Supplementary Material

gkac1138_Supplemental_File
