# Supplementary material for: Hierarchical DNA branch assembly-encoded fluorescent nanoladders for single-cell transcripts imaging
Source: Nucleic Acids Res. 2022 Dec 7;51(3):e13. doi: 10.1093/nar/gkac1138 (PMC9943671; doi:10.1093/nar/gkac1138)
Supplement: gkac1138_Supplemental_File [file gkac1138_supplemental_file.pdf]

Hierarchical DNA Branch Assembly-Encoded Fluorescent Nanoladders for Single-Cell Transcripts Imaging

Xiaowen Cao, Feng Chen*, Jing Xue, Yue Zhao, Min Bai and Yongxi Zhao*

Institute of Analytical Chemistry and Instrument for Life Science, The Key Laboratory of Biomedical Information Engineering of Ministry of Education, School of Life Science and Technology, Xi'an Jiaotong University

Xianning West Road, Xi'an, Shaanxi 710049 (China)

E-mail: yxzhao@mail.xjtu.edu.cn

fengchencf@mail.xjtu.edu.cn

Table of Contents

Table S1. The sequences used *in vitro* and *in situ* verification of RNA-primed RCA **P3**

Table S2. The codebook of 9 transcripts and corresponding primary branched barcodes sequences **P5**

Table S3. The sequences of branched barcodes and fluorescent probes **P7**

Table S4. The sequences of primers for RT-qPCR **P10**

Table S5. The detection efficiency of our method based on RT-qPCR data **P11**

Figure S1. Electrophoretic analysis of prepared circular DNAzyme probes and their RCA feasibility **P12**

Figure S2. Fluorescent analysis of RCA amplicons initiated by different primers **P13**

Figure S3. Real-time fluorescent analysis of RCA with different DNA polymerases **P14**

Figure S4. The specificity of linear DNAzyme in cleaving RNA **P15**

Figure S5. Analysis of *in situ* circularization of linear DNAzyme in our method **P16**

Figure S6. Analysis of programmable assembly of DNA barcode duplexed with different number of DFPs (labelled with Alexa 488) **P17**

Figure S7. Analysis of disturbance of branched DNA barcodes with each other **P18**

Figure S8. Investigation of FRET effect between fluorescent probes **P19**

Figure S9. The fluorescence intensity and normalized intensity of detection channels **P20**

Figure S10. One detection spectral channel of fluorescent nanoladders for RNA imaging **P21**

Figure S11. Two detection spectral channels of fluorescent nanoladders for RNA imaging **P22**

Figure S12. Representative images for multiplexed transcripts in HeLa cells with two imaging rounds **P23**

Table S1. The sequences used *in vitro* and *in situ* verification of RNA-primed RCA.

Name	Sequences (5′-3′)	
Padlock	P-AATTCACAAGCAGTGTCAGCGACTCGAAAATTC AATGTAGACAGACGTCGCGTATAATAATGCTGGGC TCTAGT	Probes for preparation of circular probe
c-padlock	CTGCTTGTGAATTACTAGAGCCCAGCA	
Pr1	GACGTCTGTCTACATTGAATT	Different primer probes
Pr2	GACGTCTGTCTACATTGAATT**G**	
Pr3	GACGTCTGTCTACATTGAATT**G-P**	
probe without DNAzyme	AATTCACAAGCAGTGTCAAAATTCAATGTAGACAG ACGTC	Probes for linear DNAzyme cleavage
linear DNAzyme	AATTCACAAGCAGTGTCAGCGACTCGAAAATTCAA TGTAGACAGACGTC	
mutated DNAzyme	AATTCACAAGCAGTAAAAGCGACAAGCCAATTCA ATGTAGACAGACGTC	
mismatch with RNA	GAAGAATCAGATCTGTCAGCGACTCGAAAATTCAA TGTAGACAGACGTC	
RNA	*UCUGUCUACAUUGAAUUGGCUGCUUGUGAAUUUU CUG*	
padlock-DNAzyme-mis	P-AATTCACAAGCAG**AAATGTCAGCGACTCGAAT AAAACAAAAACAAAACAAAG**GCGTATAATAATGC TGGGCTCTAGT	Probes for preparation of circular DNAzyme
padlock-DNAzyme	P-AATTCACAAGCAGTGTCAGCGACTCGAAAATTC AATGTAGACAGACGTCGCGTATAATAATGCTGGGC TCTAGT	
c-padlock	CTGCTTGTGAATTACTAGAGCCCAGCA	
RNA	*UCUGUCUACAUUGAAUUGGCUGCUUGUGAAUUUU CUG*	Different RNA probes
RNA-variant	*UCUGUCUACAUUGAAUUGG**GAU**CU**GA**U**UCU**U**CUG** **AA**G*	
RNA-SNV	*UCUGUCUACAUUGAAUUG**A**CUGCUUGUGAAUUUU CUG*	
P-GAPDH	P-GTACTTTATTGATGGTA TGACAAGGTGCGGCTCTGAGCCTTCCTCGGTACG GTCTGTA	Probes for RNA-primed RCA in fixed cells
P-GAPDH-mismatch	P-GTACTTTATTGATGGTA **AGTGCGAACTACTAC**CTGAGCCTTCCTCGGTACG GTCTGTA	
P-GAPDH-DNAzyme	P-GTACTTTATTGATGGTA TGTCAGCGACTCGAA TGACAAGGTGCGGCTCTGAGCCTTCCTCGGTACG GTCTGTA	

Name	Sequences (5′-3′)	
P-GAPDH-DNAzyme-mismatch	P-GTACTTTATTGATGGTA TGTCAGCGACTCGAA**AGTGCGAACTACTAC**CTGAGCCTTCCTCGGTACGGTCTGTA	
c-pGAPDH	TCAATAAAGTACTACAGACCGTACC	
Primer	CTTGTCAGTAGTAGTTCGCACT	
FP-cy5	Cy5-TGAGCCTTCCTCGGTACGGTCTGTA	
P-TK1	CTACTGTACTTTCTAATCAGCCGTCTGCTCCCCCAATCACCTTGTCAGCGACTCGAAACCTCCTTCTCTGTGCCGAGATCTATCATCATCTCAATTCTG	
P-PFN1	CTACTGTACTTTCTAATCAGCCTAAGGGGTATGGGGTAATGGTGTCAGCGACTCGAACAAAAAATAAAATGGTTTGTATCTATCATCATCTCAATTCTG	
P-CFL1	CTACTGTACTTTCTAATCGACCCTTGCAATTCATGCTTGATCTGTCAGCGACTCGAATGTCAGCTTCTTCTTGATGGATCTATCATCATCTCAATTCTG	
P-THBS1	CTACTGTACTTTCATTACGACCTCCTTCTCCCTGGAAATATGTGTCAGCGACTCGAACAAGGGATGGGGTAAAACAGATCTATCATCATCTCAATTCTG	
P-THBS1-mismatch	A**G**TATGTGTCAGCGACTCGAACAAGGGATGGGGTAAAACAGATCTATCATCATCTCAATTCTG	
P-THBS1-random	CTACTGTACTTTCATTACGACC**AGTTCTCCGCTGTGTTAGAT**TGTCAGCGACTCGAAC**GGCATCAATCGAATCTGAT**ATCTATCATCATCTCAATTCTG	Probes for preparetion of different circular DNAzyme
P-THBS1-ligation	GGTAAAACAGATCTATCATCATCTCAATTCTGCTACTGTACTTTCATTACGACCTCCTTCTCCCTGGAAATATGTGTCAGCGACTCGAACAAGGGATGG	
P-mouse-THBS1	CTACTGTACTTTCATTACGACCTCCTTCTCCCTGGAAATATGTGTCAGCGACTCGAACAAGGGGTGGTGTGGGCCTTATCTATCATCATCTCAATTCTG	
P-KI67	CTACTGTACTTTCTAATCGACCCCCGCTCCTTTTGATAGTAATGTCAGCGACTCGAAAGGCGTCTCGTGGGCCACATATCTATCATCATCTCAATTCTG	
P-ER	CTACTGTACTTTCTAATCGACCGGAGGGTCAAATCCACAAAGTGTCAGCGACTCGAATGGCACCCTCTTCGCCCAGTATCTATCATCATCTCAATTCTG	
P-PR	CTACTGTACTTTCTAATCGACCGGCTGGCTTCTGAATCCGGCTGTCAGCGACTCGAACAGGTAGTTGAGATAGGGCGATCTATCATCATCTCAATTCTG	
P-HER2	CTACTGTACTTTCTAATCGACCTGGCAGGCCAGGCCCTCGCCTGTCAGCGACTCGAACACACTCGTCCTCTGGCCGGATCTATCATCATCTCAATTCTG	

Name	Sequences (5′-3′)
P-PRKCA	CTACTGTACTTTCTAATCGACCTCATCCCTTGATGG
	ATAAGTTGTCAGCGACTCGAAATAGAGCAGTGACC
	CACAGTATCTATCATCATCTCAATTCTG
P-EGFR	CTACTGTACTTTCTAATCGACCCTCACTTACAACTG
	ACCTGCTGTCAGCGACTCGAACATCGATCATCTGC
	TTCTTGATCTATCATCATCTCAATTCTG
C-Padlock	AAGTACAGTAGCAGAATTGAGA

Table S2. The codebook of 9 transcripts and corresponding primary branched barcodes sequences.

Name	Codebook	Branched barcode 1	Branched barcode 2
TK1		GCAGATTCCGCTACGCTCCGA<br>TTCGATCAACAGCCGTCTGCT<br>CCCCCAATCACCTTGTCATCT<br>GTTTGACGCGCTGGACGGTG<br>GATTCGT	AAGTTCAACGGGACCGTGG<br>CCGATGTTTCGTCGAAACCT<br>CCTTCTCTGTGCCGAGATCT<br>ACGGCCGAATTACATCCGTC<br>GTAATCGAGGC
PFN1	10，10	GCAGATTCCGCTACGCTCCGA<br>TTCGATCAACAGCCTAAGGG<br>GTATGGGGTAATGGTGTCATC<br>TGTTTGACGCGCTGGACGGT<br>GGATTCGT	AAGTTCAACGGGACCGTGG<br>CCGATGTTTCGTCGAACAAA<br>AAATAAAATGGTTTGTATCT<br>ACGGCCGAATTACATCCGTC<br>GTAATCGAGGC
CFL1	10，5	GCAGATTCCGCTACGCTCCGA<br>TTCGATCAACAGCCCTTGCAA<br>TTCATGCTTGATCTGTCATCTG<br>TTTGACGCGCTGGACGGTGG<br>ATTCGT	AAGTTCAACGGGACCGTGG<br>CCGATGTTTCGTCGAATGTC<br>AGCTTCTTCTTGATGGATCT<br>ACGGCCGAATTACATCCGTC<br>GTAATCGAGGC
THBS1	10，1	GCAGATTCCGCTACGCTCCGA<br>TTCGATCAACAGCCTCCTTCT<br>CCCTGGAAATATGTGTCATCT<br>GTTTGACGCGCTGGACGGTG<br>GATTCGT	AAGTTCAACGGGACCGTGG<br>CCGATGTTTCGTCGAACAAG<br>GGATGGGGTAAAACAGATCT<br>ACGGCCGAATTACATCCGTC<br>GTAATCGAGGC
ER	5，10	GCAGATTCCGCTACGCTCCGA<br>TTCGATCAACAGCCGGAGGG<br>TCAAATCCACAAAGTGTCATC<br>TGTTTGACGCGCTGGACGGT<br>GGATTCGT	AAGTTCAACGGGACCGTGG<br>CCGATGTTTCGTCGAATGGC<br>ACCCTCTTCGCCCAGTATCT<br>ACGGCCGAATTACATCCGTC<br>GTAATCGAGGC
PR	5，5	GCAGATTCCGCTACGCTCCGA<br>TTCGATCAACAGCCGGCTGG<br>CTTCTGAATCCGGCTGTCATC<br>TGTTTGACGCGCTGGACGGT<br>GGATTCGT	AAGTTCAACGGGACCGTGG<br>CCGATGTTTCGTCGAACAGG<br>TAGTTGAGATAGGGCGATCT<br>ACGGCCGAATTACATCCGTC<br>GTAATCGAGGC
Her2	5，1	GCAGATTCCGCTACGCTCCGA<br>TTCGATCAACAGCCTGGCAG<br>GCCAGGCCCTCGCCTGTCATC<br>TGTTTGACGCGCTGGACGGT<br>GGATTCGT	AAGTTCAACGGGACCGTGG<br>CCGATGTTTCGTCGAACACA<br>CTCGTCCTCTGGCCGGATCT<br>ACGGCCGAATTACATCCGTC<br>GTAATCGAGGC
KI67	1，10	GCAGATTCCGCTACGCTCCGA<br>TTCGATCAACAGCCCCCGCTC<br>CTTTTGATAGTAATGTCATCTG<br>TTTGACGCGCTGGACGGTGG<br>ATTCGT	AAGTTCAACGGGACCGTGG<br>CCGATGTTTCGTCGAAAGGC<br>GTCTCGTGGGCCACATATCT<br>ACGGCCGAATTACATCCGTC<br>GTAATCGAGGC

Name	Codebook	Branched barcode 1	Branched barcode 2
EGFR	1，5	GCAGATTCCGCTACGCTCCGATTCGATCAACGACCCTCACTTACAACTGACCTGCTGTCATCTGTTTGACGCGCTGGACGGTGGATTCGT	AAGTTCAACGGGACCGTGGCCGATGTTTCGTCGAACATCGATCATCTGCTTCTTGATCTACGGCCGAATTACATCCGTCGTAATCGAGGC
PRKCA	1，1	GCAGATTCCGCTACGCTCCGATTCGATCAACGACCTCATCCCTTGATGGATAAGTTGTCATCTGTTTGACGCGCTGGACGGTGGATTCGT	AAGTTCAACGGGACCGTGGCCGATGTTTCGTCGAAATAGAGCAGTGACCCACAGTATCTACGGCCGAATTACATCCGTCGTAATCGAGGC

Table S3. The sequences of branched barcodes and fluorescent probes.

Name	Sequences (5′-3′)	
Branched barcode1-1	TTGATCGAATCGGAGCGTAGCGGAATCTGCTTACAGACCGGCTCAAGAAG	Branch DNA barcodes
Branched barcode1-2	TTGATCGAATCGGAGCGTAGCGGAATCTGCTTACAGACCGGCTCAAGAAGTTACAGACCGGCTCAAGAAG	
Branched barcode1-3	TTGATCGAATCGGAGCGTAGCGGAATCTGCTTACAGACCGGCTCAAGAAGTTACAGACCGGCTCAAGAAGTTACAGACCGGCTCAAGAAG	
Branched barcode1-4	TTGATCGAATCGGAGCGTAGCGGAATCTGCTTACAGACCGGCTCAAGAAGTTACAGACCGGCTCAAGAAGTTACAGACCGGCTCAAGAAGTTACAGACCGGCTCAAGAAG	
Branched barcode1-5	TTGATCGAATCGGAGCGTAGCGGAATCTGCTTACAGACCGGCTCAAGAAGTTACAGACCGGCTCAAGAAGTTACAGACCGGCTCAAGAAGTTACAGACCGGCTCAAGAAGTTACAGACCGGCTCAAGAAG	
Branched barcode1'-1	TTACAGACCGGCTCAAGAAGACGAATCCACCGTCCAGCGCGTCAAACAGA	
Branched barcode1'-2	TTACAGACCGGCTCAAGAAGTTACAGACCGGCTCAAGAAGACGAATCCACCGTCCAGCGCGTCAAACAGA	
Branched barcode1'-3	TTACAGACCGGCTCAAGAAGTTACAGACCGGCTCAAGAAGTTACAGACCGGCTCAAGAAGACGAATCCACCGTCCAGCGCGTCAAACAGA	
Branched barcode1'-4	TTACAGACCGGCTCAAGAAGTTACAGACCGGCTCAAGAAGTTACAGACCGGCTCAAGAAGTTACAGACCGGCTCAAGAAGACGAATCCACCGTCCAGCGCGTCAAACAGA	
Branched barcode1'-5	TTACAGACCGGCTCAAGAAGTTACAGACCGGCTCAAGAAGTTACAGACCGGCTCAAGAAGTTACAGACCGGCTCAAGAAGTTACAGACCGGCTCAAGAAGACGAATCCACCGTCCAGCGCGTCAAACAGA	
Branched barcode2-1	CGAAACATCGGCCACGGTCCCGTTGAACTTAGAGAGTAGTTAGTCGCACT	
Branched barcode2-5	CGAAACATCGGCCACGGTCCCGTTGAACTTAGAGAGTAGTTAGTCGCACTAGAGAGTAGTTAGTCGCACTAGAGAGTAGTTAGTCGCACTAGAGAGTAGTTAGTCGCACTAGAGAGTAGTTAGTCGCACT	
Branched barcode2'-1	AGAGAGTAGTTAGTCGCACTGCCTCGATTACGACGGATGTAATTCGGCCG	
Branched barcode2'-5	AGAGAGTAGTTAGTCGCACTAGAGAGTAGTTAGTCGCACTAGAGAGTAGTTAGTCGCACTAGAGAGTAGTTAGTCGCACTAGAGAGTAGTTAGTCGCACTGCCTCGATTACGACGGATGTAATTCGGCCG	
FP-488	/Alexa488/AGTGCGACTAACTACTCTCT	Fluorescent probes

Name	Sequences (5′-3′)
FP-cy5	/Cy5/CTTCTTGAGCCGGTCTGTAA
FP-555	/Alexa555/CTCAATTCTGCTACTGTACT

Table S4. The sequences of primers for RT-qPCR.

Name	Sequences (5′-3′)
Forward THBS1 primer	GCCACAGTTCCTGATGGAG
Reverse THBS1 primer	CCATGGAGACCAGCCATC
Forward Her2 primer	CTTTGCTGTCCTGTTCACCA
Reverse Her2 primer	TCATCATCTTCACATTGAGTAGGC
Forward PR primer	CTTTGCATGTCTGTCCAACC
Reverse PR primer	CTGCTCTAGTCTTACCCGTGTG

Table S5. The detection efficiency of our method based on RT-qPCR data.

Transcript	RNA-primed RCA (copy number/per cell)	*Ct* values	RT-qPCR (copy number/per cell)	Detection efficiency
THBS1	26.96	25.75	94.25	28.6%
Her2	8.16	27.59	27.13	30.1%
PR	2.52	30.83	8.58	29.3%

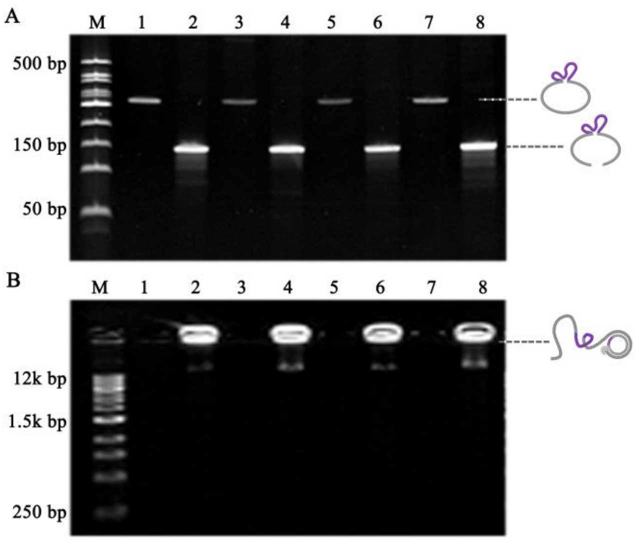

Figure S1. Electrophoretic analysis of prepared circular DNAzyme probes and RCA feasibility of them. (A) Analysis of padlock probes and prepared circular DNAzyme. Lane 1, 3, 5, 7: circular DNAzymes; Lane 2, 4, 6, 8: padlock probes. The probes were resolved by 15% denaturing PAGE. (B) 1% agarose gel electrophoresis of amplicon using prepared circular DNAzymes with primer (lane 2, 4, 6, 8) and without primer (Lane 1, 3, 5, 7).

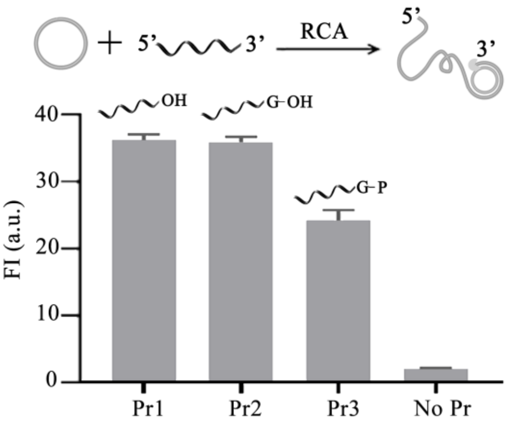

Figure S2. Fluorescent analysis of RCA amplicons initiated by different primers. Pr1: the primer without 3' modifications; Pr2: the primer with a base overhang; Pr3: the primer with both a base overhang and 3'-phosphate.

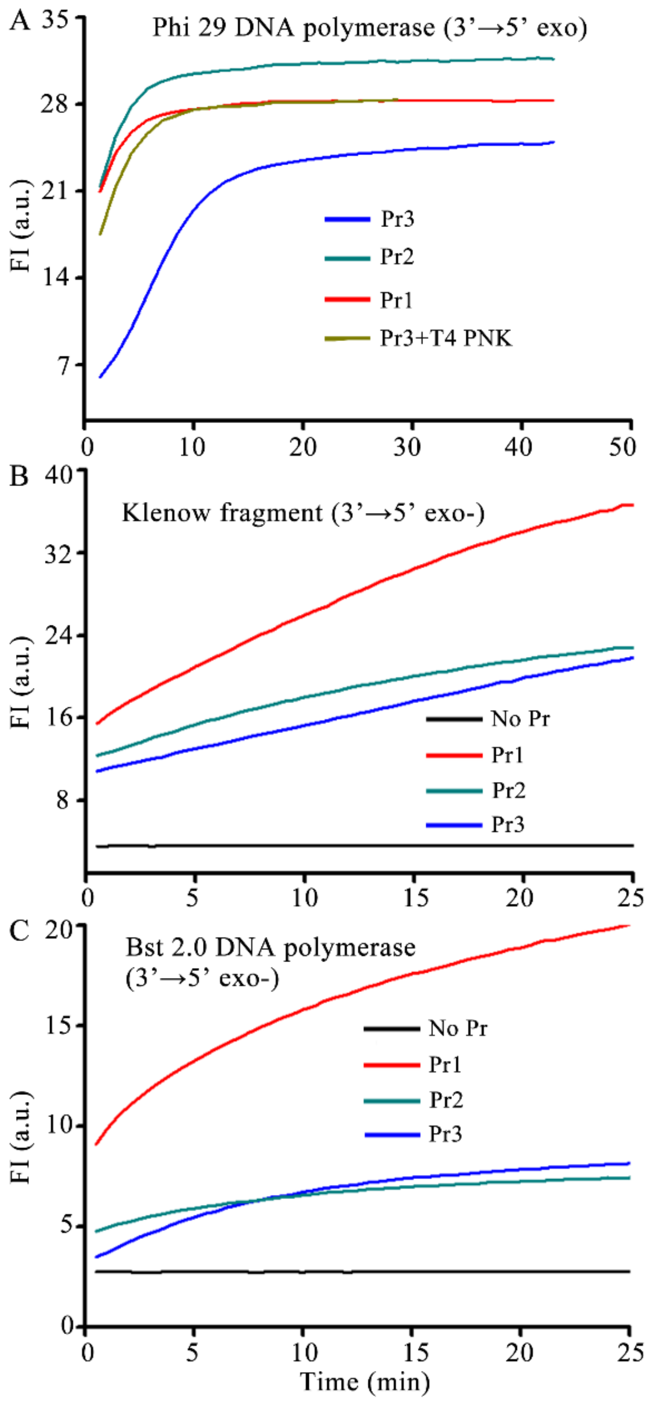

Figure S3. Real-time fluorescent analysis of RCA with different DNA polymerases.

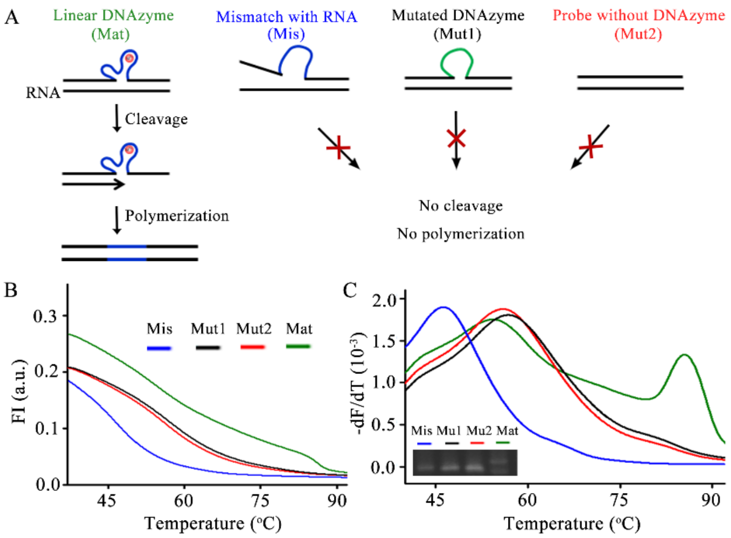

Figure S4. The specificity of linear DNAzyme in cleaving RNA. (A) Schematic representation of cleavage and polymerization using different probes. (B) The real-time melting of products from A. (C) The derivation of B. Inset: agarose gel electrophoresis of different products from A.

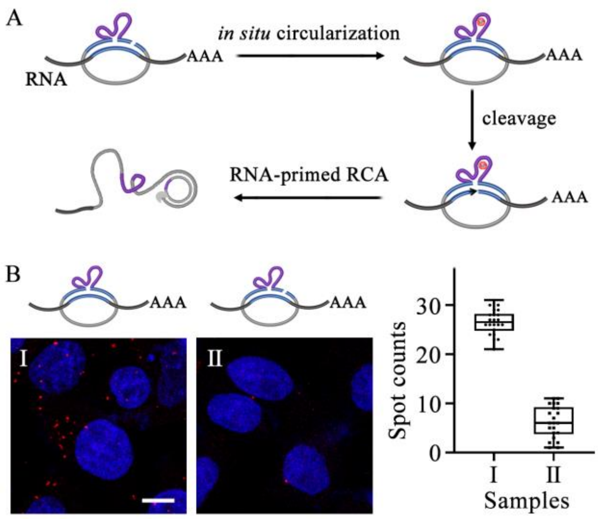

Figure S5. Analysis of *in situ* circularization of linear DNAzyme in our method. (A) Schematic overview of the experiment process. (B) Left: representative images of target RNA in single cells using the RCA-primed RCA and additional *in situ* circularization followed by RCA-primed RCA (red, Alexa 555 for THBS1; blue, DAPI; Hela cells were used here). The scale bar is 10 µm. Right: statistical analysis of amplicon spot counts for each sample in single cells (N=20). N represents the number of single cells for each sample.

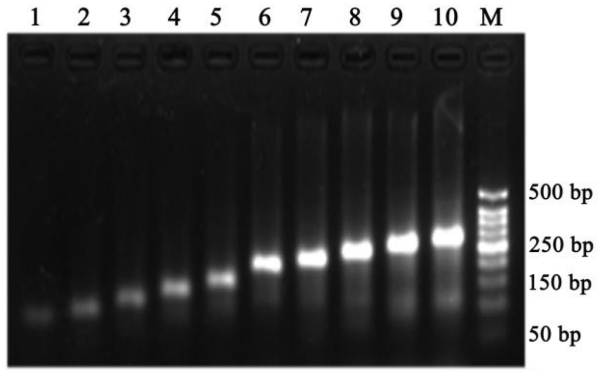

Figure S6. Analysis of programmable assembly of DNA barcode duplexed with different number of DFPs (labelled with Alexa 488). The assemblies were resolved by 3.5% agarose gel. Lane1-10: the number of DFP is 1, 2, 3, 4, 5, 6, 7, 8, 9, 10. The M means 50bp DNA ladder.

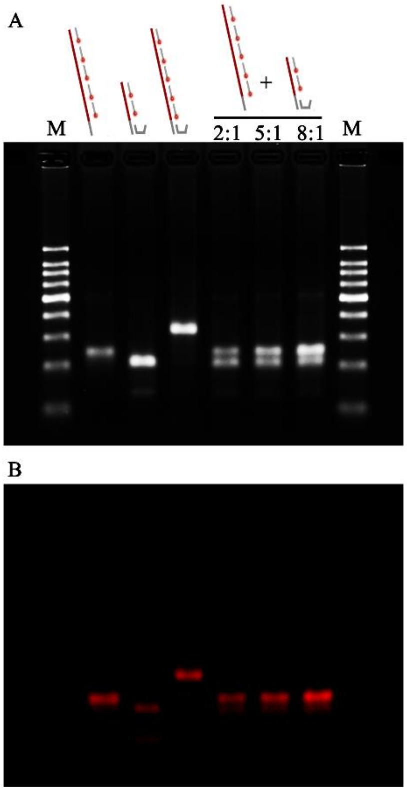

Figure S7. Analysis of disturbance of branched DNA barcodes with each other. Substrates and products were resolved by 3.5% agarose gel. (A) The ultraviolet imaging of gel. The M means 50bp DNA ladder. (B) The fluorescent imaging of gel in (A). Red: Cy5.

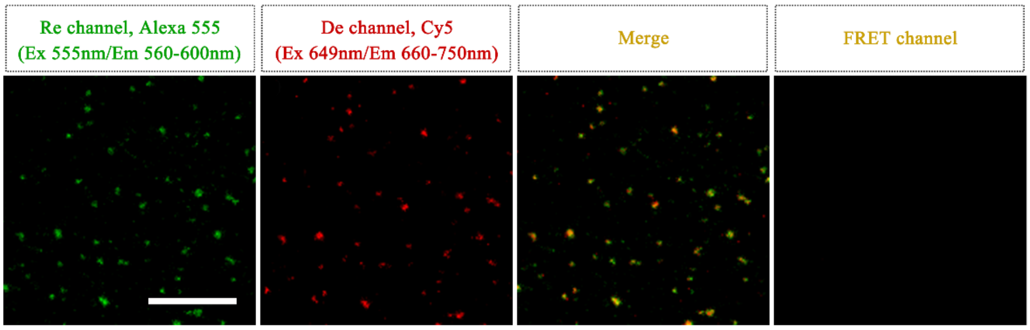

Figure S8. Investigation of FRET effect between fluorescent probes. The fluorescence image of RCA amplicons in Alexa555 channel, Cy5 channel, Alexa555-Cy5 merged channel and FRET channel. The scale bar is 10 μm.

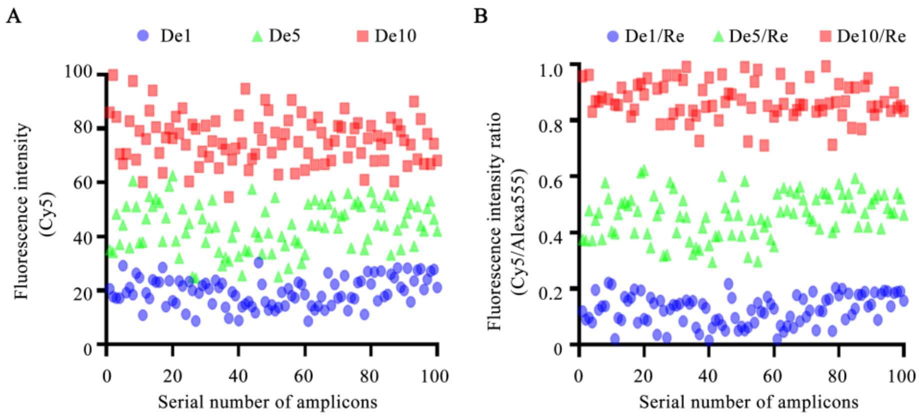

Figure S9. The fluorescence intensity and normalized intensity of detection channels (the fluorescence intensity of detection channel was normalized by dividing that of reference channel).

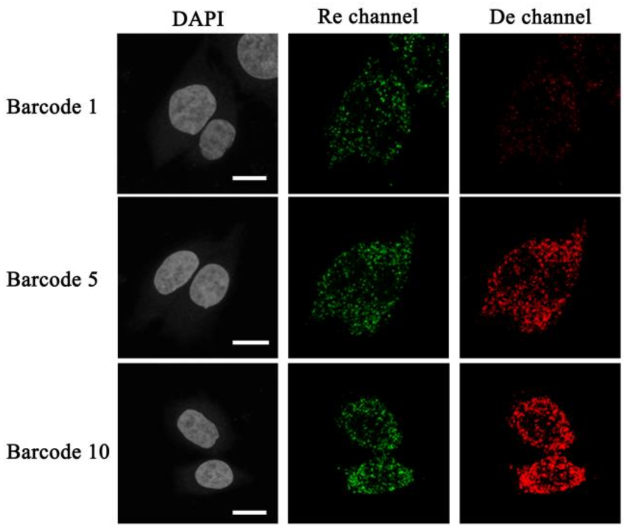

Figure S10. One detection spectral channel of fluorescent nanoladders for RNA imaging. The fluorescence image of TK1 in MCF-10A in different channels, the scale bars are 10 μm.

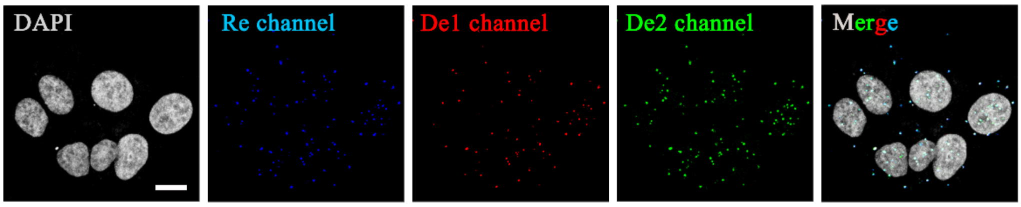

Figure S11. Two detection spectral channels of fluorescent nanoladders for RNA imaging. The fluorescence image of CFL1 in MCF-10A in different channels, the scale bar is 10 μm.

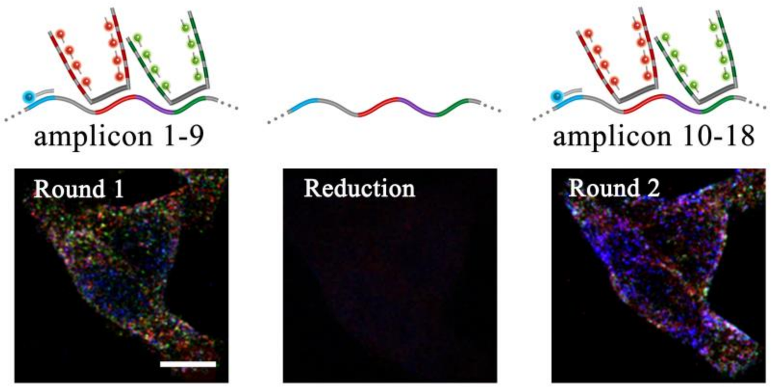

Figure S12. Representative images for multiplexed transcripts in HeLa cells with two imaging rounds. The image after reduction by 60% formamide demonstrates efficient removal of fluorescent signals between rounds. The scale bar is 10 μm.
